# Effect of the Mechanical Load on the Carbonation of Concrete: A Review of the Underlying Mechanisms, Test Methods, and Results

**DOI:** 10.3390/ma14164407

**Published:** 2021-08-06

**Authors:** Zhiyuan Liu, Philip Van den Heede, Nele De Belie

**Affiliations:** Magnel-Vandepitte Laboratory for Structural Engineering and Building Materials, Ghent University, 9052 Ghent, Belgium; zhiyuan.liu@ugent.be (Z.L.); philip.vandenheede@ugent.be (P.V.d.H.)

**Keywords:** concrete, carbonation, mechanical load, crack, loading frame

## Abstract

As one of the major causes of concrete deterioration, the carbonation of concrete has been widely investigated over recent decades. In recent years, the effect of mechanical load on carbonation has started to attract more attention. The load-induced variations in crack pattern and pore structure have a significant influence on CO_2_ transport which determines the carbonation rate. With different types of load, the number, orientation, and position of the induced cracks can be different, which will lead to different carbonation patterns. In this review paper, the carbonation in cracked and stress-damaged concrete is discussed first. Then, literature about the effect of sustained load during carbonation is compared in terms of load type and load level. Finally, the advantages and disadvantages of possible test methods for investigating the effect of sustained load on carbonation are discussed with respect to loading devices, load compensation, and specimen size.

## 1. Introduction

Carbonation of concrete is one of the major causes of reinforced concrete deterioration. Concrete is inevitably porous. The pores and microcracks exist both at the inside and on the surface of concrete, which allows the penetration of CO_2_ when the surface of concrete is exposed to the atmosphere. In the presence of moisture, CO_2_ will dissolve in the pore solution and form ${\mathrm{HCO}}_{3}^{-}$ and ${\mathrm{CO}}_{3}^{2-}$ ions, and consequently cause carbonation of hydration products of concrete such as portlandite (Ca(OH)_2_) [1], calcium silicate hydrates (C-S-H) [2,3,4], and ettringite [5,6], even of unhydrated cement particles [7,8] such as tricalcium silicate (C_3_S) and dicalcium silicate (C_2_S). In the carbonation process, different polymorphs of CaCO_3_ are formed, including calcite, vaterite, and aragonite. At high alkalinity, the Ca^2+^ mainly originates from the dissolution of portlandite. With the continuous consumption of Ca(OH)_2_, the alkalinity of concrete decreases gradually. When the carbonation front reaches the reinforcement, further carbonation will cause the depassivation of steel reinforcement. From then on, there is an onset of active corrosion, which shortens the service life of reinforced concrete structures. Therefore, as a critical issue, carbonation of concrete has been widely investigated [9,10,11,12].

The carbonation process is dominated by several factors including material composition, carbonation condition, and curing. These key factors determine the carbonation rate and carbonation products. Therefore, numerous efforts have been made to understand the CO_2_ transport and reaction mechanisms [13,14,15]. Whereas, only limited research exists about the carbonation of concrete under mechanical load. Reinforced concrete is designed to bear mechanical load which usually causes variation in porosity and crack patterns. According to Eurocode 2 [16], the design concrete strength is 67% of characteristic strength at most. In some cases, the limitation of stress in concrete is even lower. For example, considering creep, the stress limitation is 0.45 of the breaking load. Though reinforced concrete structures have been calculated to endure service loads without critical impacts on their mechanical properties over the service life, loads may make concrete more vulnerable to the ingress of water, CO_2_, chloride, and other agents. Therefore, when investigating the deterioration process of reinforced concrete under environmental effects, the effect of mechanical load should not be ignored. For decades, researchers have gained a preliminary understanding of the effect of precracking on the carbonation of concrete [17,18,19]. The issue of the carbonation under sustained load is more realistic and should therefore receive considerable critical attention. Service life prediction models can be optimized using the output (e.g., critical threshold load levels) of accelerated carbonation tests under sustained load.

In this paper, it is first discussed how cracks and stress damage induced by external mechanical load affect the carbonation resistance (Section 2). Next, the effects of sustained loads throughout the service life are discussed in view of the expected gas permeability and carbonation depth (Section 3). In addition, a literature survey was included on currently available dedicated experimental setups for investigating concrete under combined mechanical load and carbonation (Section 4). A critical comparison between the different test setups has been made. A lot of attention went into the evaluation of their feasibility, reliability, advantages, and disadvantages in relation to relevant modes and levels of loading.

## 2. Carbonation in Cracked and Load-Damaged Concrete

Essentially, when investigating the effect of load on carbonation, the most critical factor is the variation in crack and pore structure caused by the application of load. With different types and levels of mechanical load applied, the cracks vary in number, length, width, and pattern. Consequently, the process of CO_2_ transport is changed to some extent. Based on the mechanisms of concrete carbonation, the CO_2_ transport and carbonation affect each other mutually. On the one hand, better CO_2_ transport properties result in a faster carbonation. On the other hand, the formation of carbonation products of portlandite, including calcite, aragonite, and vaterite, results in a volume increase of 11.2%, 2.9%, and 18.7%, respectively [20]. The produced water and precipitated CaCO_3_ in Portland cement concrete will fill the pores and cracks and therefore lower the CO_2_ transport properties in turn.

### 2.1. Effect of Crack Pattern on Concrete Carbonation

Cracks are very common in concrete. Even without external load, the heat of hydration, autogenous and drying shrinkage, and improper curing may cause cracks in concrete surfaces. In reinforced concrete beams, concrete in the flexural tension zone will be cracked once in service. These cracks, as flow channel for CO_2_, may facilitate direct exposure of the rebars to CO_2_. Hence, the carbonation takes place at the interface between steel rebar and concrete even though only limited carbonation occurs at the concrete cover [17,21], which means the concrete cover no longer has an effect at the location of the crack. However, only wide enough cracks, acting as additional exposure surfaces to CO_2_, will have a great impact on carbonation. While microcracks would only accelerate the carbonation slightly. Small cracks may close again as a result of carbonation-induced autogenous healing [22]. To gain a better understanding of the latter aspect, some researchers investigated the carbonation behavior of concrete in which cracks with certain geometries were induced deliberately. In some cases, a lower crack width threshold was found, below which CO_2_ transport is not increased in comparison with uncracked material. On the other hand, an upper crack width threshold may occur, above which the carbonation rate remains stable independent of the crack width. The results obtained in the literature are summarized in Table 1.

In some studies, artificial cracks are induced by positioning a thin plate inside the specimens during casting and removing this plate after hardening of the specimens. Via this method, standardized cracks without tortuosity are created. The crack depth and crack width can be easily controlled that way. Though the crack width determines the amount of CO_2_ molecules entering into the crack per unit of time, the following CO_2_ transport along the crack path also depends on the carbonation at the crack walls which consumes CO_2_. If the CO_2_ transport into the crack is slow, there can be a concentration gradient of CO_2_ along the crack depth. Consequently, the carbonation rate is the highest at the outer surface, followed by the crack surface and the lowest at the crack tip [23]. Different authors observed an upper crack width threshold of 100 μm at CO_2_ levels of 1% [25], 3% [27], and 20% [23]. The carbonation rate remains stable as the crack width increases above this value. At a CO_2_ level of 50%, Alahmad found that the carbonation depth measured at an artificial crack surface with a crack width of 10 μm is already higher than the carbonation depth in an uncracked region of the concrete surface [26], indicating a lower crack width threshold below 10 µm. A possible explanation is that a much higher CO_2_ concentration causes adequate CO_2_ supply even in very fine cracks. Besides, an empirical formula was developed by De Schutter to quantify the effect of crack depth and width on the carbonation [24].

However, real cracks in concrete are much more complicated than artificial cracks. The differences between artificial cracks and realistic cracks should be carefully considered. For instance, the surfaces of artificial cracks are smoother and higher in cement content (the so-called “wall effect”) [24]. Moreover, the width of realistic cracks becomes gradually lower along the crack path. The interlocking and tortuosity of realistic cracks will hinder the transport of CO_2_. Generally, artificial cracks cause a higher carbonation depth. So, the effect of realistic cracks should also be investigated. In some studies, realistic cracks are induced through the application of flexural or splitting load while the crack width is being monitored. Contrary to artificial cracks, it is found that the carbonation rate still increases when the realistic crack width exceeds 100 μm (CO_2_ level: 1% and 5%) [25,28]. Under a much higher CO_2_ level of 50%, Alahmad [26] found that carbonation rate perpendicular to a realistic crack wall is similar to that at the concrete surface when the crack width is higher than 60 μm, and that CO_2_ transport appears to stop for crack width of 9 μm or less.

Much work has also been carried out on the gas permeability of cracked concrete. In view of the great impact of the crack pattern on gas transport, any factors which influence the load-induced crack pattern, such as aggregate type, aggregate size [31], and mix proportion [32], can more or less influence the gas permeability and also carbonation. For instance, under 3-point bending, the cracks may generate inside light-weight aggregates and can be even wider than in the matrix, which results in a higher carbonation rate than for normal-weight aggregate concrete [27]. Nevertheless, under a compressive load, the stiffness of light-weight aggregate is more closely matching the stiffness of the mortar matrix than in the case of normal-weight concrete. So, light-weight aggregate concrete appeared to be capable of enduring a higher level of compressive stress without forming continuous crack channels that could allow the gas flow through them [33]. Due to the divergent test regimes, the effect of these factors can be complicated. The difficulty of quantitatively and accurately characterizing the crack patterns complicates a well-founded discussion.

Furthermore, attention should be paid to the difference between gas permeability and carbonation. The sensitivity of a gas permeability test and a carbonation test to the connected macrocracks are different. It was found that the laminar-turbulent gas flow transition always occurs at a certain range of gas permeability and inlet gas pressure [34]. The cracks convey the larger part of the gas flow. The water saturation degree no longer affects the gas permeability measurement for cracked concrete [34,35], which is not the case for carbonation.

### 2.2. Effect of Load-Induced Damage on Concrete Carbonation

#### 2.2.1. Static Load

**Figure 1:**
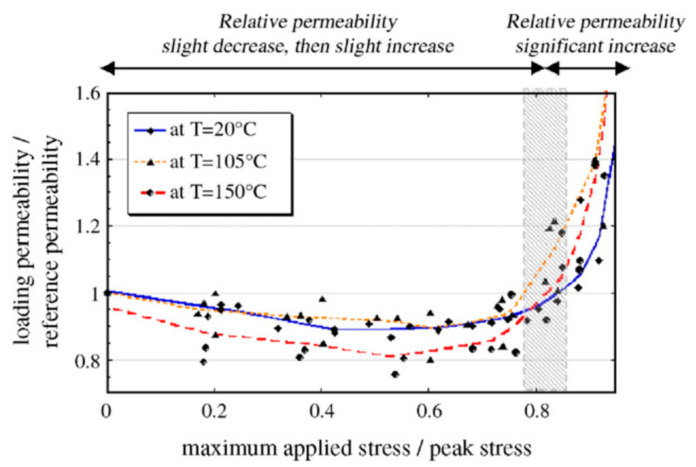
Effect of compressive stress damage on permeability. Copied from reference [40] with copyright permission from Elsevier.

Apparently, the carbonation rate increases as the degree of damage increases [36]. A higher degree of damage means more cracks, which then accelerate the CO_2_ transport. However, a higher external mechanical load not always results in a higher degree of damage. For example, the compaction effect under a relatively low compressive load causes the partial closure of cracks and pores. As shown in Figure 1, due to the contracting volumetric behavior, the permeability of concrete slightly decreases. As the load level increases, new cracks are generated and microcracks coalesce. The volumetric response becomes dilating, and the permeability increases, first slightly and then significantly from 80% of the peak stress onwards. Similar results are reported regarding the carbonation of concrete, uniaxial compressive load will decrease the carbonation rate when the stress level is below a threshold load level of 0.7 [37]. On the contrary, damage caused by tension leads to an increase in carbonation rate [38]. When the cement is replaced by different types of supplementary cementitious materials, tension will cause different extents of acceleration of the carbonation. Ground granulated blast-furnace slag makes the carbonation rate rise faster when concrete is in tension, compared with fly ash [39].

#### 2.2.2. Fatigue Load

Apart from being exposed to static loads, reinforced concrete structures are also subject to fatigue load during the service life. Good examples of this are bridges and pavements. The applied cyclic loading will cause a progressive growth of internal microcracks which initiate from inherent flaws. When a microcrack initiates, it keeps on growing during each loading cycle until a critical size is reached. Eventually, this results in a significant increase in irrecoverable strain [41] and ultimately even in failure of the concrete structure. Certainly, these microscopic internal changes increase CO_2_ transport properties and are responsible for a detrimental effect on the durability of concrete. However, the way it influences carbonation is complicated. As the porosity of concrete linearly increases with the tensile fatigue damage, there is a plateau phase between two rapid growing phases at a low and high damage degree whereby the carbonation rate increases slightly with the damage degree [42].

Jiang [43] found that the carbonation rate of concrete was more sensitive to tensile fatigue damage than to compressive fatigue damage. Eccentric fatigue load which causes a load gradient and damage gradient in concrete does not significantly influence the way relative humidity, temperature, and CO_2_ concentration affect the carbonation process in concrete. Zhang [36] found that the carbonation rate increased after specimens were loaded several times longitudinally and transversely (compressive load level: 0.3–0.5 of breaking load), even though there were no visible cracks on the surface of the concrete. Dai [44] found that, though the carbonation rate increased after cyclic compressive load, the 28 days strength after 10,000 cycles was higher than the 28 days strength after 5000 cycles. Extra microcracks induced by more fatigue load cycles seem to boost the autogenous healing effect attributable to carbonation.

## 3. Carbonation of Concrete under Sustained Load

Indeed, load-induced damage of concrete is a problem that engineers and scientists may encounter. Attention should also be paid to sustained loads which will be present during the entire service life of a concrete structure. The effect of mechanical load on carbonation cannot be simply considered as stress damage because of the difference in microstructure before and after loading. In the studies about stress damage, specimens are loaded following a certain loading protocol for a short time and unloaded before being put in the CO_2_ chamber. Nevertheless, the crack patterns under load and after releasing the load are not the same. For example, if the designed loading level does not exceed the ultimate splitting stress, the crack opening displacement may reduce by 80% after unloading [45]. Wan [37] found that the specific crack area started to decrease significantly at the 0.25 stress level during the unloading process, which shows an obvious deformation recovery caused by cracks closure. Moreover, the permeability of concrete measured after being unloaded was higher than during compressive loading when at low and intermediate stress level, while it was inverse when a high compressive stress level (0.8 of breaking load at 20 ℃) was applied [40].

In view of the differences in crack pattern and pore structure between the loaded and unloaded situation, more researchers have started to investigate the effect of sustained load on the carbonation of concrete recently, not merely precracking and stress damage. Though the studies on carbonation under sustained load are more realistic, it should be noted that creep of concrete can have a great influence in case of accelerated carbonation in a laboratory environment. Creep of concrete is noticeably higher at early age. Therefore, if the specimens were put into a CO_2_ chamber right after the load application, the substantially higher creep at early stage can influence the CO_2_ transport. Further studies should pay attention to the early-stage creep in combination with accelerated carbonation in a CO_2_ chamber.

When different types and levels of external load are applied, the cracks vary in number, length, width, and pattern, and the pore structure is also changed. A denser concrete structure will slow down the diffusion of CO_2_ while less dense concrete structures contain more pores and cracks which act as flow channels of CO_2_ and accelerate the diffusion of CO_2_. A larger crack not only makes CO_2_ diffusion easier, but also provides more room for carbonation product formation. With different types of load, distinct results of carbonation depth can be obtained. Due to the difficulties encountered for maintaining a dynamic load inside a CO_2_ chamber, generally a static load is applied throughout the whole experiment, either compressive load, tensile load, flexural load, or splitting load. The effect of different types and levels of mechanical load is discussed with respect to gas permeability and carbonation depth. A summary of representative literature discussed in this paper is shown in Table 2.

### 3.1. Compressive and Tensile Load

The effect of applied compressive load on a concrete structure highly depends on the stress level. Lim [60] investigated the crack patterns under different stress levels through microscopy observation. According to his research on concrete crack length after compressive load, bond cracks occur at the aggregate–mortar interface when the stress level is above 0.3 times the failure stress and the total crack length increases significantly when the stress level exceeds 0.5. Notable isolated mortar cracks occur at a stress level of 0.7 and interconnect with bond cracks at the 0.9 stress level. Though the crack opening displacement reduces after the load is completely removed [45], it is helpful to understand the transport properties of concrete under sustained compressive load.

**Figure 2:**
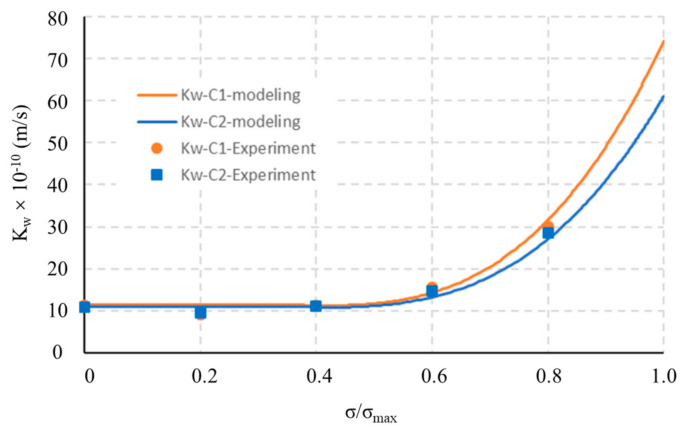
Water permeability versus compressive level for two concrete mix proportion (experimental and modeling results). Copied from reference [69] with copyright permission from Elsevier.

When the stress level reaches a certain threshold, crack growth goes to another stage and the crack pattern changes greatly. A threshold stress level for permeability tests on concrete is also identified by other researchers. According to the results of water permeability experiments, Banthia [61,62] found that the threshold of sustained compressive stress for both plain concrete and fiber-reinforced concrete appears to be approximately 0.3 times the failure stress. A slight decrease was also reported at 0.2 times the failure stress, as shown in Figure 2. In addition to mix proportion, the exact threshold also depends on the age and loading history [63]. Wimalasiri developed a numerical method based on the concrete damage plasticity model to determine the permeability under load. It was found that a higher grade of concrete, larger aggregate diameter, larger ITZ thickness, and sharper aggregate angle will result in a lower threshold [64]. The increase in temperature also leads to a lower threshold [65].

Tang found that the gas permeability of unloaded specimens is higher than for specimens at a 0.3 stress level and lower than for specimens at a 0.6 stress level [66], which shows that a threshold for gas permeability may also occur at the 0.3–0.6 stress level. Both the gas and water permeability of concrete decrease as the sustained compressive load increases when the load level is below the threshold, while the permeability increases significantly after exceeding the threshold. The densification of concrete under a relatively low compressive load is the main reason for this phenomenon. The intrinsic concrete cracks generated before the permeability test will be partially closed due to the external stress and the pore structure is compressed. Besides, the densification effect is influenced by the direction of the sustained compressive load. Cracks perpendicular to the compressive load close while the cracks parallel to the compressive load tend to open. Therefore, a confining pressure shows a stronger densification effect because the fluid flow is perpendicular to the compressive load. In this case, the whole side surface of the cylindrical specimen is under compression and the expansion of concrete is only along the axial direction. When the confining pressure is only 6% of ultimate uniaxial compressive strength, the water permeability of uniaxial compressive load damaged concrete can decrease by 69%. Through nuclear magnetic resonance, it is found that the 0.1–1000 μm mesopores are dominant before the application of confining pressure, while the micropores smaller than 0.1 μm become dominant after the application of confining pressure [67]. Nevertheless, the permeability is less sensitive to confining pressure for sound concrete specimens [68].

**Figure 3:**
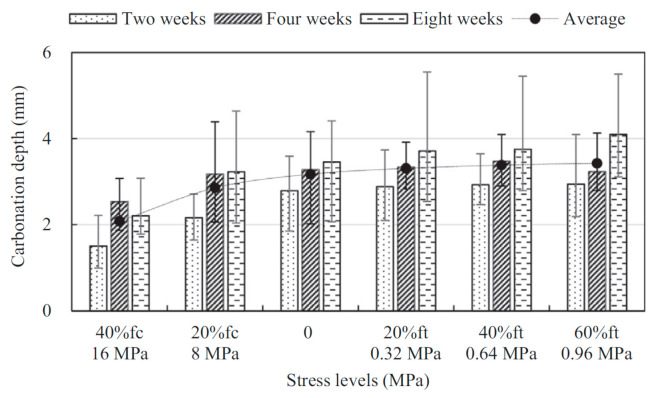
Typical effect of uniaxial compressive and tensile load on carbonation rate. Copied from reference [46] with copyright permission from Elsevier.

The typical effect of uniaxial compressive and tensile load on carbonation rate is shown in Figure 3. The threshold for carbonation under sustained compressive load seems to be higher than the threshold for gas and water permeability. When comparing with unloaded specimens, the carbonation rate under sustained compressive load is still lower when 0.4 [46], 0.5 [47,48], and even 0.75 [49] of the failure stress is reached. On the one hand, the pressure during a gas permeability test is much higher than the atmospheric pressure during the carbonation test, which will absolutely cause a discrepancy in the results. On the other hand, the densification effect of compressive load can be underestimated for gas permeability. With the increase in compressive load, the pores and matrix are compacted continuously, while cracks start developing. When only a small number of cracks are generated, gas can permeate through and the obtained gas permeability rises, but the CO_2_ still needs to penetrate into the greatly densified matrix and react with the hydration products for carbonation to occur. Moreover, carbonation itself may play an important role. The formation of carbonation products caused by the chemical reaction between CO_2_ and hydration products can block the path of CO_2_ transport, which has a great impact on CO_2_ transport properties in turn. Therefore, although the gas permeability test is less time-consuming, for evaluation of the effects of load on carbonation, an actual carbonation test will provide more reliable results.

However, some researchers also reported a threshold for carbonation under sustained compressive load at a stress level of around 0.2–0.3 [50,51,52,53], which is similar to the results for permeability. Interestingly, the size of the specimens in their study was much smaller than the size in the previously described research (usually 100 × 100 × 400 mm^3^). In the first two cases [50,51], the specimens were 100 mm high in the direction of compressive load. These sizes are similar to specimens used in permeability tests and are usually not large enough to provide a uniform distribution of compressive stress. With a lower specimen height, because of the effect of horizontal confinement, more cracks are generated and they only occur in a thin outer layer [70]. In the last two cases, the sizes of the specimens are large (100 × 100 × 300 mm^3^ in [52] and 100 × 100 × 400 mm^3^ in [53]), but they are hollow in the middle (hollow core of 25 and 40 mm in diameter, respectively) so that the rebar can pass through to provide a compressive load. Furthermore, this may cause a great difference in crack pattern. Therefore, the specimen size plays an important role in these cases and makes the threshold occur at a lower stress level.

On the contrary, a uniaxial tensile load only results in the generation and growth of microcracks perpendicular to the direction of the tensile load and makes concrete less dense. Hence, uniaxial tensile load always leads to an increase in gas permeability [66] and carbonation rate [53]. Recycled aggregate concrete is more sensitive to tensile load as opposed to compressive load because of larger microstructural changes under tensile load [71]. Besides, though different patterns of cracks are induced, a splitting tensile load has a similar effect on concrete carbonation.

One of the most significant purposes of studying carbonation under sustained load is to optimize service life prediction models for design of reinforced concrete structures. In the presence of tensile loads, service life prediction based on an experimental evaluation of sound concrete can lead to an overestimation. For compressive load, the existence of the threshold value can also cause errors in the service life prediction. Moreover, the compressive load threshold greatly varies depending on how the concrete microstructure responds to the mechanical load, in terms of crack propagation and pore structure changes. It is essential to determine the compressive load threshold for concrete structures that are more prone to intrinsic flaws such as light-weight aggregate concrete (weak ITZ and low aggregate strength), recycled aggregate concrete (with weak ITZ), and 3D printed concrete (with anisotropy caused by the weak interlayer bonding). Apart from avoiding the overestimation of the service life, the densification effect of the compressive load has the potential to improve the durability by hindering the ingress of the aggressive substances to some extent.

### 3.2. Flexural Load

Considering that few reinforced concrete structures are serving under uniaxial tensile stress in practice, three-point bending and four-point bending modes are used in many studies for a better understanding of concrete carbonation in practice. Besides, the loading frame for applying a bending load is usually smaller than a loading frame for applying uniaxial tensile loads, and can easily be put in a CO_2_ chamber.

However, it should be pointed out that the system is more complicated when bending load is applied. Firstly, for a specimen under bending load, there exists a bending tensile zone and a bending compressive zone at the same time which are divided by the neutral plane. For both bending compressive zone and bending tensile zone individually, the CO_2_ transport behavior is similar to that under uniaxial load. However, the transport properties under compressive and tensile load, determined by pore structure and crack pattern, are totally different and should be investigated separately. Even so, because the compressive strength of concrete is much higher than the tensile strength, when the load level is set according to the breaking bending load, the actual compressive load in the compressive zone is very low. Thus, the threshold of compressive load probably cannot be observed. Secondly, the flexural shear stress may also cause a change in crack pattern. Castel [72] found that the natural carbonation depth in the lateral surface (close to tensile surface and mainly under tensile stress) is always deeper than in the tensile surface. He ascribes this to surface moisture deposit and the effect of gravity which hinder the transport of CO_2_. Besides, there exists a gradient of stress in the direction perpendicular to the neutral plane. Thus, CO_2_ transport along this direction actually does not occur under the same stress level. This should be taken into consideration when proposing a new theoretical formula for carbonation depth prediction.

Similar to the results of carbonation depth when uniaxial tensile load is applied, the carbonation rate in the bending tensile zone always increases with the increase in stress level [73]. Obviously, when the stress level is increased, the tensile stress in the bending tensile zone becomes higher and leads to the generation of more microcracks. CO_2_ transport increases in the less dense concrete matrix and, therefore, the carbonation rate rises. However, for the bending compressive zone, as mentioned before, the threshold probably cannot be observed when the load level is set according to the breaking bending load. In some studies, the carbonation rate is still decreasing with stress levels up to 0.5 [54,55,56,57], which is caused by the compaction effect of the external compressive stress. Nevertheless, some researchers reported a threshold over which a significant increase in carbonation rate was observed. With smaller specimens (70.7 × 70.7 × 212 mm^3^) under four-point bending, Zheng [58] found a threshold at around a stress level of 0.2. Based on a CaCO_3_ profile obtained through titration, Wan [59] found that the threshold is around 0.3. Due to the great differences in mix proportion, specimen size, and test method, it is hard to conclude which is the main factor for the threshold through the comparison of literature, and further research regarding load-induced cracks and pore structure variation should be carried out. Based on the results of carbonation depths under mechanical load in existing literature, Wang [74] proposed empirical formulas to describe how compressive stress level and tensile stress level influence carbonation rate. Several studies have focused on service life prediction and carbonation modelling considering the effects of flexural load [75,76,77].

## 4. Test Methods for Carbonation of Concrete under Sustained Load

When investigating the effect of damage on carbonation, usually no special apparatus is required, and there is less restriction when designing and conducting the experiment. An expected degree of damage can be obtained through the control of hydraulics. However, the experiments in which sustained loads are involved are more complicated. A stable load should be maintained on specimens during long-term exposure to certain environmental factors. Due to the lack of a standard test method, several different test apparatus and test procedures have been designed to achieve different types of sustained load.

### 4.1. Specimen Size and Loading Devices

In view of the limitation of the CO_2_ chamber size, the size of the specimens should not be taken too large. Meanwhile, as discussed in Section 3.1, specimen size has a significant effect on the crack pattern which determines the carbonation rate. With a low specimen height, because of the effect of horizontal confinement, the cracks may only occur in a very thin outer layer whereas the remainder of the specimen remains uncracked [70]. Therefore, the specimens should be kept large enough to avoid uneven distribution of coarse aggregate and cracks which are generated during the loading. In most studies, prismatic specimens have been adopted, for example, 100 × 100 × 400 mm^3^ [53], 100 × 100 × 300 mm^3^ [78] and 40 × 40 × 160 mm^3^ [46,79].

**Figure 4:**
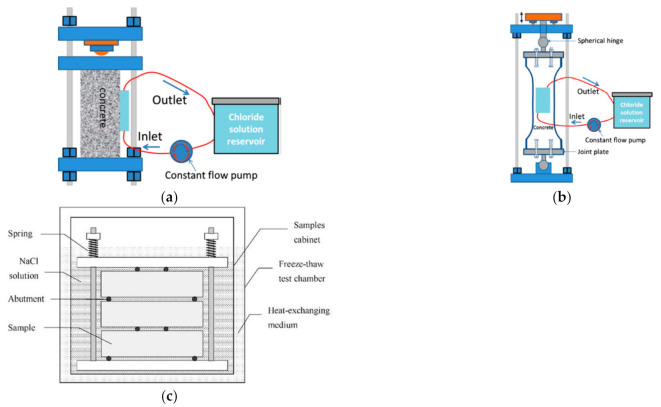
Loading frames developed for applying different types of load. (**a**) Loading frame for applying uniaxial compressive load; (**b**) Loading frame for applying uniaxial tensile load and (**c**) Loading frame for applying 4-point bending load. (**a**,**b**) are copied from reference [81] with copyright permission from Springer Nature. (**c**) is copied from reference [82] with copyright permission from Elsevier.

The sustained loads are applied and maintained with loading frames which are similar to the post-tensioning system for prestressed concrete. Usually, threaded bar tendons which are put inside or outside of the specimens are used so that both tensile and compressive stress can be provided. When investigating the effect of load on concrete under different environmental conditions other than carbonation, the loading frame for applying external mechanical load is mostly the same. Thus, the loading frames for environmental action other than carbonation are also included in the following discussion. For applying different types of loads including uniaxial compressive load [46,51], uniaxial tensile load [80,81], three-point bending load [72], and four-point bending load [79,82] by use of external bar tendons, several loading frames have been designed. Typical loading frames are shown in Figure 4a–c. Such a loading frame with external threaded bar tendons can provide a more even and accurate load through adjusting four nuts. A loading frame which simulates a corbel and applies flexural loads was reported [83].

**Figure 5:**
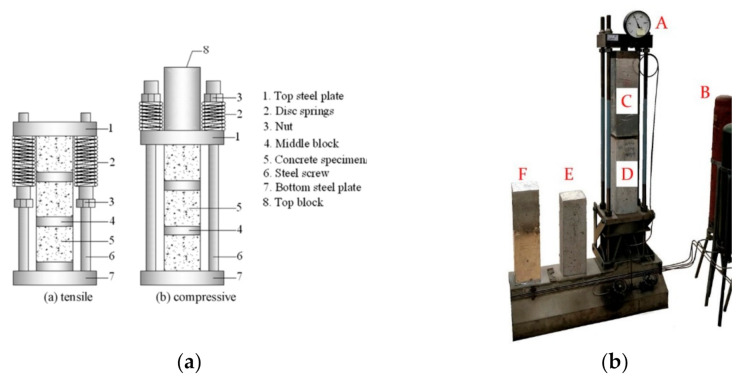
Loading frames which can compensate the relaxed stress. (**a**) Loading frame with disc spring and (**b**) Creep setup. (a) is copied from reference [50] with copyright permission from Elsevier. (b) is copied from reference [84] with copyright permission from the author.

Based on the loading setups mentioned above, some researchers added springs on the bar tendons, as shown in Figure 4c. In this case, the load is transmitted through the springs and not merely through the bar tendons. So, when inevitable stress losses occur, the applied external load will not decrease too much because of the buffering effect of the spring. Nevertheless, to provide large enough load within a slight strain, very strong springs are required, which will greatly increase the total weight and size of the setup. If the springs are not strong enough, the applied stress cannot reach the designed stress level [46], especially for compressive stress. In order to keep the total weight as light as possible and allow for a compact design, disc springs (Belleville washer), which can support very large loads with a small installation space, are used [50], as shown in Figure 5a. Moreover, if the CO_2_ chamber is large enough, the creep setup is certainly an ideal option for applying uniaxial compressive load. For example, as shown in Figure 5b, the external load is provided by the gas pressure of the compressed air in a gas bottle. The load is more stable because the decrease in gas volume caused by the slight deformation of concrete has a smaller effect on the load level.

**Figure 6:**
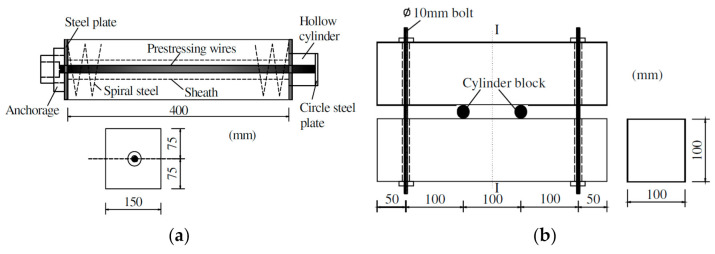
Loading frames with internal bar tendons. (**a**) Loading frame with internal bar tendon and (**b**) Loading frame with internal bar tendon. Copied from reference [86] with copyright permission from American Society of Civil Engineers.

Still, the main shortcoming of external bar tendons is that the loading frame may be too large and heavy, which makes it difficult to manually transport it and install it into the CO_2_ chamber, especially for the loading frames in which springs are added. Therefore, an alternative can be to use internal unbonded bar tendons. As shown in Figure 6a,b, a circular duct is precast in the concrete for the bar tendon. Obviously, loading with internal bar tendons greatly reduces both the size and weight of the loading setup. However, the accuracy and stability of the load for these setups still need further investigation. In addition, since the size of specimens and load setups are limited by the dimensions of the CO_2_ chamber, the prescribed carbonation environment can only be maintained at part of the outer surface of the specimens [85], especially for very large concrete specimens. However, maintaining the exposure conditions is more difficult in terms of CO_2_ concentration, relative humidity, and temperature.

### 4.2. Load Application and Compensation

For loading frames with external bar tendons, hydraulics and torque wrenches [30] can be used to apply load. Applying load through hydraulics is more accurate but the operator should pay attention to the stress losses. If the load applied by the hydraulics is kept at a fixed value when tightening the nuts, the load carried by the concrete specimen can drop greatly after the hydraulics is removed because of the bolts loosening and the elastic deformation of the loading frame. If the hydraulics is kept stationary when tightening the nuts, the load applied by the hydraulic machine decreases gradually, and the load on the concrete specimens reaches the design value when the load applied by the hydraulics drops to zero. By this means, the stress loss occurring right after the removal of the hydraulics can be avoided because the loading frame, including the bolts and nuts, is already loaded. However, the nuts have to be tightened by wrench manually, which may be infeasible if a compressive load is to be applied. The load can also be applied by torque wrench but is less accurate. The reason is that the measured torque is derived from the friction between the nut and screw which highly depends on the nut and screw specifications and even whether they are free of rust and lubricated or not. Yet, it is still a simple way to apply a relatively low load. Moreover, the operator can apply the load with a wrench manually by checking the resulting strain when the strain gauges are glued on the specimens.

During carbonation, the load maintained by the loading frame always drops with time. This phenomenon of stress relaxation is inevitably caused by shrinkage and creep of concrete and bolt loosening. When the designed stress level is relatively low, regularly releasing the load followed by reloading would be a solution to the problem. However, it is reported that, when the load level is over 54% of the failure load, the apparent Poisson’s ratio starts to change clearly, which means that irreversible plastic deformation occurs [37]. In this case, reloading will result in unexpected changes in the crack pattern. Besides, some researchers applied excessive load on concrete specimens to counter the stress loss, which is similar to the procedure for prestressed concrete [86,87]. In the case of prestressed concrete, usually slightly excessive load (3–5% of designed load) is applied to ensure the structural safety. However, this can only keep the average stress near or above the design value but not mitigate the effect of shrinkage and creep. Since there is no requirement for structural safety in a loading setup for laboratory tests, more measures are needed to avoid too much stress loss during the carbonation. For instance, as mentioned before, the stress relaxation during the long-term carbonation experiment can be alleviated by the designed springs. In addition, the load can be compensated by regularly tightening the nuts when the load is monitored by a load cell. Since the load cell is costly and will make the design of the loading frame more complicated, tightening the nuts to keep the length of springs can be an alternative.

## 5. Conclusions

Carbonation of concrete is always one of the major focus points when investigating the durability of reinforced concrete. Many efforts have been made to understand the carbonation process in sound concrete. However, during the service life, it is nearly impossible for reinforced concrete to be under only one deteriorating action. Reinforced concrete is designed to bear mechanical load. So, the deterioration process of concrete under environmental effects is actually always coupled with mechanical load. Therefore, over the past decade, researchers have started to investigate the effect of mechanical load on the carbonation of concrete.

This paper gives an overview of how mechanical load influences the carbonation of concrete from different perspectives, depending on the type of load and the induced crack pattern. Moreover, it is discussed how a dedicated experimental setup is achieved for investigating the effect of different types of sustained load. The key observations can be summarized as follows.

Carbonation rate increases rapidly as the cracks width increases, while it remains stable after a certain crack width threshold is exceeded (around 100 μm for artificial cracks).

Compressive load can densify concrete and slow down the carbonation slightly at a low load level. The carbonation rate significantly increases once a certain threshold is reached because of the generation and connection of cracks. However, tensile loads always result in a higher porosity of concrete and increase in carbonation rate.

Future research should be carried out to gain a deeper understanding of the coupled effect of mechanical load and carbonation.

The key factors for the threshold of compressive load remain uncertain when looking at existing research. The values given in different papers show a large discrepancy. More efforts should be made to determine the threshold so that acceleration of carbonation can be possibly avoided or taken into account in service life design.

There is a great difference in crack pattern and pore structure when mechanical load is applied using different protocols. It is of a great importance to quantitatively characterize the cracks and then establish a relation with the carbonation rate.

Efforts should be made to gain a deeper understanding of the carbonation mechanism under mechanical load. Since carbonation can also change the microstructure and therefore affect the mechanical properties of concrete, the response of the carbonated and non-carbonated concrete to the mechanical load will normally not be the same. Furthermore, the carbonation process changes the load-damaged microstructure as well. The mutual microstructural effects of the mechanical load (especially sustained load) and carbonation should be addressed in more depth in future research.

In view of the difficulties in the test setups for the carbonation under sustained load, the gas permeability can be a good alternative. However, the reported threshold values for gas permeability are generally much lower than the threshold value for carbonation depth. The relation between the gas permeability and carbonation under sustained load needs to be further established.

## Figures and Tables

**Table 1:** Overview of literature evaluating effect of cracks on carbonation.

Crack Type	Crack Inducing Method	CO_2_ Level	Crack Pattern	Lower/Upper Crack Width Threshold (μm)	Reference
Artificial crack	Embedding of thin plates	20%	100, 200, 300, 500, 620 μm in width and 20 mm in depth	−/100	[23]
Artificial crack	Embedding of thin plates	10%	200, 300, 500 μm in width and 5, 10 mm in depth	*	[24]
Artificial crack	Embedding of thin plates	1%	100, 200, 300 μm in width and 30 mm in depth	−/100	[25]
Realistic crack	Splitting	1%	100, 200, 300 μm in width	*	[25]
Artificial crack	Sawed and smoothed	50%	10–150 μm in width	*	[26]
Realistic crack	Expansive core	50%	9–400 μm in width	9/60	[26]
Artificial crack	Embedding of thin plates	3%	100, 200, 300 μm in width and 10 mm in depth	−/100	[27]
Realistic crack	Bending	3%	100, 150 μm in width	*	[27]
Realistic crack	Bending	5%	50–350 μm in width	*	[28]
Realistic crack	Bending	Natural	80, 500, 700 μm in width	*	[29]
Realistic crack	Bending	2%	50, 100, 150, 200, 300 μm in width	*	[30]

* the carbonation depth keeps increasing with the increase in crack width under the given condition.

**Table 2:** Overview of research about the effect of sustained load on carbonation.

Specimen Size (mm^3^)	CO_2_ Level	Load Type	Load Level (Compression/ Tension)	Carbonation Depth Variation (under Compression/Tension) ^1^	Compressive Load Level Threshold	Reference
40 × 40 × 160	5%	Uniaxial compression and tension	0.2, 0.4/0.2, 0.4, 0.6		*	[46]
100 × 100 × 400	20%	Compression and bending tension	0.2, 0.35, 0.5/0.3, 0.5, 0.7	0.61/1.38	*	[47]
100 × 100 × 400	20%	Compression and bending tension	0.05, 0.3, 0.5/0.3, 0.5, 0.7	0.21/1.27	*	[48]
100 × 100 × 400	20%	Compression and bending tension	0.15, 0.3, 0.45, 0.6, 0.75	0.534/1.30	*	[49]
100 × 100 × 50	20%	Uniaxial compression and tension	0.2, 0.4, 0.6	0.91–1.45/1.39	0.2	[50]
100 × 100 × 40 for compression and φ100 × 40 for tension	5%	Compression and splitting tension	0.3, 0.6	0.88–1.26/1.44	0.3	[51]
100 × 100 × 300 for compression and 100 × 100 × 500 for bending	20%	Compression and bending tension	0.3, 0.5, 0.7, 0.9		0.3	[52]
100 × 100 × 400 for compression and 100 × 170 × 440 for tension	20%	Uniaxial compression and tension	0.35, 0.65, 0.75/0.35, 0.65		0.35	[53]
100 × 100 × 400	20%	4-point bending	0.3, 0.4, 0.5		*	[54]
100 × 100 × 300	20%	3-point bending	0.2, 0.3, 0.4	0.61/1.51	*	[55]
100 × 100 × 400	20%	4-point bending	0.3, 0.4, 0.5		*	[56]
100 × 100 × 550	20%	4-point bending	0.2, 0.4, 0.6	0.72/2.28	*	[57]
70.7 × 70.7 × 212	20%	4-point bending	0.2, 0.4, 0.6	0.89–1.26/1.66	0.2	[58]
100 × 100 × 400	20%	4-point bending	0.3, 0.5, 0.6, 0.8	0.89–1.51/1.64	0.3	[59]

Note: the load level is the ratio of the applied load to the breaking load; the compressive load level threshold means the threshold above which carbonation rate sharply increases with the load level; * means no threshold is observed; ^1^ means the range of the ratio of the carbonation depths under different compressive/tensile load levels to the carbonation depths without load.

## Data Availability

Data sharing not applicable.
